# (*E*)-2-{[4-(Di­methyl­amino)­benzyl­idene]amino}-5-nitro­phenol

**DOI:** 10.1107/S160053681400871X

**Published:** 2014-04-26

**Authors:** Yousef Hijji, Ray J. Butcher, Jerry P. Jasinski

**Affiliations:** a Chemistry Department, Morgan State University, 1700 East Cold Spring Lane, Baltimore, MD 21251, USA; b Department of Chemistry, Howard University, 525 College Street NW, Washington, DC 20059, USA; c Department of Chemistry, Keene State College, Keene, NH 03410, USA

## Abstract

The title Schiff base compound, C_15_H_15_N_3_O_3_, crystallizes with two mol­ecules (*A* and *B*) in the asymmetric unit. Each mol­ecule adopts an *E* conformation around the C= N imine bond. The two mol­ecules have minor differences in their conformations. In mol­ecule *A*, the dihedral angle between the nitro group and its benzene ring is 2.1 (2)° and that between the two benzene rings is 0.88 (7)°, while the corresponding angles for mol­ecule *B* are 5.7 (1) and 2.45 (6)°, respectively. In each mol­ecule, there is an intra­molecular O—H⋯N hydrogen bond. In the crystal, inversion-related mol­ecules are linked *via* O—H⋯O hydrogen bonds forming *A*–*A* and *B*–*B* dimers. These dimers are linked *via* C—H⋯O hydrogen bonds involving the nitro O atoms, forming *A*–*A*–*A* and *B*–*B*–*B* slabs that lie parallel to one another and to (010).

## Related literature   

For related structures, see: Rodríguez *et al.* (2012 ▶); Valkonen *et al.* (2012 ▶); Gül *et al.* (2007 ▶); Reyes *et al.* (2004 ▶); Hijji *et al.* (2014 ▶). For the applications of Schiff bases as anion sensors, see: Hijji *et al.* (2009 ▶), and in non-linear optics, see: Muñoz *et al.* (2008 ▶).
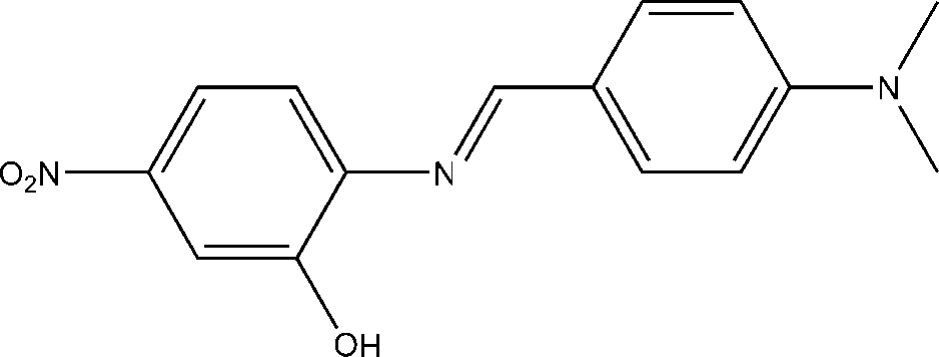


## Experimental   

### 

#### Crystal data  

C_15_H_15_N_3_O_3_
*M* *_r_* = 285.30
Triclinic, 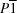

*a* = 6.1435 (3) Å
*b* = 14.3844 (8) Å
*c* = 15.8516 (9) Å
α = 108.038 (5)°
β = 91.258 (4)°
γ = 96.033 (4)°
*V* = 1322.37 (13) Å^3^
*Z* = 4
Cu *K*α radiation
μ = 0.84 mm^−1^
*T* = 123 K
0.32 × 0.24 × 0.19 mm

#### Data collection  

Agilent Xcalibur (Ruby, Gemini) diffractometer
Absorption correction: multi-scan (*CrysAlis PRO*; Agilent, 2012 ▶) *T*_min_ = 0.938, *T*_max_ = 1.000
9056 measured reflections
9056 independent reflections
7791 reflections with *I* > 2σ(*I*)
*R*_int_ = 0.034

#### Refinement  

*R*[*F* ^2^ > 2σ(*F* ^2^)] = 0.041
*wR*(*F* ^2^) = 0.119
*S* = 1.04
9056 reflections
392 parameters
H atoms treated by a mixture of independent and constrained refinement
Δρ_max_ = 0.32 e Å^−3^
Δρ_min_ = −0.21 e Å^−3^

### 

Data collection: *CrysAlis PRO* (Agilent, 2012 ▶); cell refinement: *CrysAlis PRO*; data reduction: *CrysAlis PRO*; program(s) used to solve structure: *SHELXS2013* (Sheldrick, 2008 ▶); program(s) used to refine structure: *SHELXL2013* (Sheldrick, 2008 ▶); molecular graphics: *SHELXTL* (Sheldrick, 2008 ▶); software used to prepare material for publication: *SHELXTL*.

## Supplementary Material

Crystal structure: contains datablock(s) I. DOI: 10.1107/S160053681400871X/su2709sup1.cif

Structure factors: contains datablock(s) I. DOI: 10.1107/S160053681400871X/su2709Isup2.hkl

Supporting information file. DOI: 10.1107/S160053681400871X/su2709Isup3.cml

CCDC reference: 997774

Additional supporting information:  crystallographic information; 3D view; checkCIF report

## Figures and Tables

**Table 1:** Hydrogen-bond geometry (Å, °)

*D*—H⋯*A*	*D*—H	H⋯*A*	*D*⋯*A*	*D*—H⋯*A*
O1*A*—H1*A*⋯N2*A*	0.85 (2)	1.99 (2)	2.5860 (16)	126 (2)
O1*A*—H1*A*⋯O1*A* ^i^	0.85 (2)	2.41 (2)	2.8738 (16)	114.7 (19)
O1*B*—H1*B*⋯N2*B*	0.84 (2)	1.993 (19)	2.5896 (16)	127.5 (19)
O1*B*—H1*B*⋯O1*B* ^ii^	0.84 (2)	2.45 (2)	2.8853 (15)	113.7 (17)
C7*B*—H7*BA*⋯O1*B* ^iii^	0.95	2.58	3.1870 (17)	122
C15*A*—H15*C*⋯O3*A* ^iv^	0.98	2.59	3.3989 (19)	141

Symmetry codes: (i) 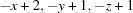; (ii) 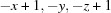; (iii) 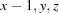; (iv) 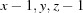.

**Fig. 1.**
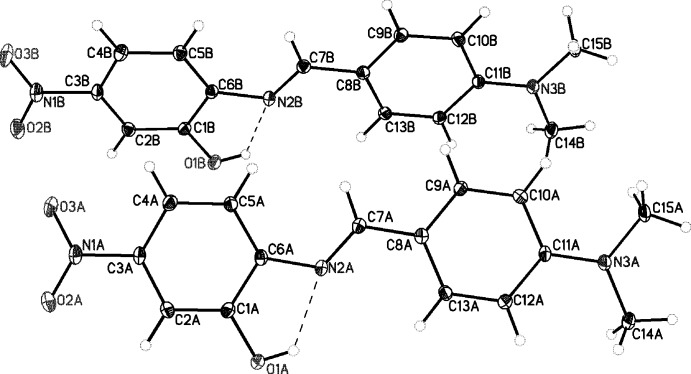
A view of the molecular structure of the two independent molecules (A and B) of the title compound, with atom labelling. The intramolecular N—H···O hydrogen bonds are shown as dashed lines (see Table 1 for details).

**Fig. 2.**
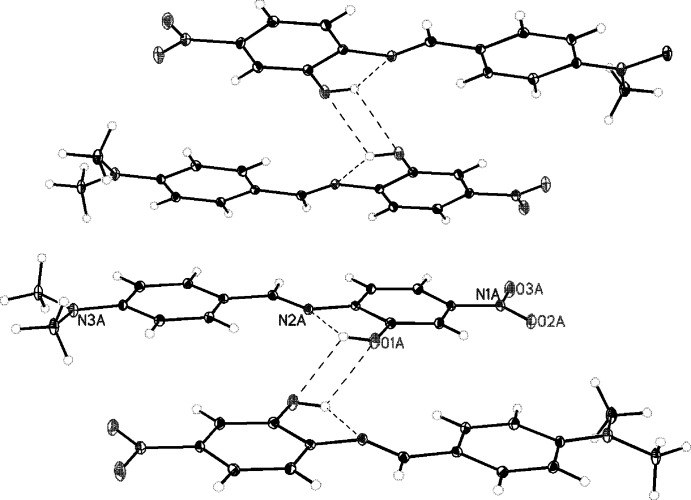
Crystal packing diagram for the title compound viewed along the a axis. The hydrogen bonds are shown as dashed lines (see Table 1 for details).

**Fig. 3.**
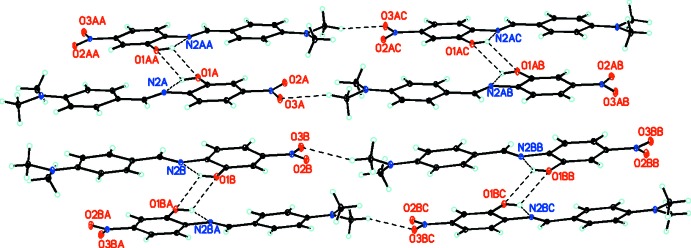
The -A-A-A- and -B-B-B- slabs that lie parallel to one another and to (010)

## supplementary crystallographic information

### 1. Comment

Schiff bases are used as ligands to form complexes with metals and borane and such complexes have application in non-linear optical dyes (Rodríguez *et al.*, 2012; Reyes *et al.*, 2004) and as anion sensors (Hijji *et al.*, 2009; Muñoz *et al.* 2008). Related structures were reported by (Gül *et al.*, 2007; Muñoz *et al.*, 2008; Valkonen *et al.*, 2012; Hijji *et al.*, 2014).

The title compound is a Schiff base prepared by the reaction of 4-di­methyl­amino­benzaldehyde with 2-amino-5-nitro­phenol under microwave conditions.It crystallized with two molecules (A and B) in the asymmetric unit, Fig. 1. Each molecule adopts an E conformation about the C=N imine bond: C7A═N2A in A and C7B═N2B in B. The two molecules in the asymmetric unit have minor differences in their conformations: In molecule A the dihedral angle between the nitro group (N1A/O2A/O3A) and its benzene ring (C1A—C6A) is 2.1 (2)° and between the two benzene rings (C1A—C6A and C8A—C13A) is only 0.88 (7)°, while for molecule B the corresponding angles are 5.7 (1)° and 2.45 (6)°, respectively. For each molecule there is an intra­molecular hydrogen bond (Table 1 and Fig. 1) involving the OH group.

In the crystal, inversion related individual molecules are linked via O—H···O hydrogen bonds forming A—A and B—B dimers (Table 1 and Fig. 2). These dimers are linked via C—H···O hydrogen bonds involving the nitro group O atoms forming -A—A—A- and -B—B—B- slabs that lie parallel to one another and to (010) - see Fig. 3.

In a related 4-nitro­phenyl derivative (Hijji *et al.*, 2014) there are no inter­molecular C—H···O hydrogen bonds involving the nitro group.

### 2. Experimental

4-dimethamino­benzaldehyde (0.150 g, 1.0 mmol) and 5-nitro-2-amino phenol (0.15 g, 1.0mmol) were placed in a Biotage microwave tube. The mixture was heated in the Biotage initiator microwave for 5 min at 393 K. Upon cooling a brown solid formed. It was dissolved in ethanol and allowed to recrystallize to provide purple crystals (0.20g, 70% yield; M.p. 495-498 K). A sample was recrystallized from ethanol by slow evaporation to provide crystals suitable for X-ray diffraction analysis. Spectroscopic data for the title compound is available in the archived CIF.

### 3. Refinement

The hydroxyl H atoms were located in a difference Fourier map and freely refined. The C-bound H atoms were placed in calculated positions and treated as riding atoms: C—H = 0.95 and 0.99 Å for CH and CH_3_ H atoms, respectively, with *U*_iso_(H) = 1.5*U*_eq_(C-methyl) and = 1.2U_eq_(C) for other H atoms.

### Crystal data

**Table:**

C_15_H_15_N_3_O_3_	*Z* = 4
*M**_r_* = 285.30	*F*(000) = 600
Triclinic, *P*1	*D*_x_ = 1.433 Mg m^−^^3^
*a* = 6.1435 (3) Å	Cu *K*α radiation, λ = 1.54184 Å
*b* = 14.3844 (8) Å	Cell parameters from 3857 reflections
*c* = 15.8516 (9) Å	θ = 5.0–74.3°
α = 108.038 (5)°	µ = 0.84 mm^−^^1^
β = 91.258 (4)°	*T* = 123 K
γ = 96.033 (4)°	Block, yellow-brown
*V* = 1322.37 (13) Å^3^	0.32 × 0.24 × 0.19 mm

### Data collection

**Table:**

Agilent Xcalibur (Ruby, Gemini) diffractometer	9056 independent reflections
Radiation source: Enhance (Cu) X-ray Source	7791 reflections with *I* > 2σ(*I*)
Graphite monochromator	*R*_int_ = 0.034
Detector resolution: 10.5081 pixels mm^-1^	θ_max_ = 74.2°, θ_min_ = 5.0°
ω scans	*h* = −7→7
Absorption correction: multi-scan (*CrysAlis PRO*; Agilent, 2012)	*k* = −17→17
*T*_min_ = 0.938, *T*_max_ = 1.000	*l* = −19→19
9056 measured reflections	

### Refinement

**Table:**

Refinement on *F*^2^	0 restraints
Least-squares matrix: full	Hydrogen site location: mixed
*R*[*F*^2^ > 2σ(*F*^2^)] = 0.041	H atoms treated by a mixture of independent and constrained refinement
*wR*(*F*^2^) = 0.119	*w* = 1/[σ^2^(*F*_o_^2^) + (0.0805*P*)^2^ + 0.1015*P*] where *P* = (*F*_o_^2^ + 2*F*_c_^2^)/3
*S* = 1.04	(Δ/σ)_max_ < 0.001
9056 reflections	Δρ_max_ = 0.32 e Å^−^^3^
392 parameters	Δρ_min_ = −0.21 e Å^−^^3^

### Special details

**Table:**

Experimental. Spectroscopic data for the title compound : ^1^H-NMR (400 MHz) δ ppm (DMSO-d6): 10.07 (s, 1H), 9.74 (s, 1H), 7.768 (d, J = 8.86 Hz, 1H), 7.68 (d, J = 8.97, 2H), 7.606 (dd, J = 8.87, 2.5 Hz, 1 H), 7.49 (d, J = 2.5 Hz, 1H), 6.78 (d, J = 8.97, 2 H), 6.63 (d, J = 8.87, 1 H), 3.069 (s, 6 H). ^13^C-NMR (DMSO-d6, 100 MHz) δ ppm: 189.82, 154.16, 145.54, 142.41, 135.46, 131.50, 124.48, 118.29, 111.12, 111.02, 108.59, 39.60.
Geometry. All e.s.d.'s (except the e.s.d. in the dihedral angle between two l.s. planes) are estimated using the full covariance matrix. The cell e.s.d.'s are taken into account individually in the estimation of e.s.d.'s in distances, angles and torsion angles; correlations between e.s.d.'s in cell parameters are only used when they are defined by crystal symmetry. An approximate (isotropic) treatment of cell e.s.d.'s is used for estimating e.s.d.'s involving l.s. planes.
Refinement. Refined as a 2-component twin.

### Fractional atomic coordinates and isotropic or equivalent isotropic displacement parameters (Å^2^)

**Table:**

	*x*	*y*	*z*	*U*_iso_*/*U*_eq_	
O1A	1.00034 (17)	0.44325 (8)	0.56033 (7)	0.0261 (2)	
H1A	0.918 (4)	0.4353 (16)	0.5142 (16)	0.045 (6)*	
O2A	1.05386 (18)	0.41323 (9)	0.86286 (8)	0.0311 (3)	
O3A	0.7307 (2)	0.35008 (10)	0.88265 (8)	0.0384 (3)	
N1A	0.8610 (2)	0.38209 (9)	0.83748 (8)	0.0239 (3)	
N2A	0.60070 (19)	0.38449 (8)	0.49667 (8)	0.0185 (2)	
N3A	0.22830 (19)	0.38081 (9)	0.11251 (8)	0.0224 (3)	
C1A	0.8619 (2)	0.41242 (10)	0.61406 (10)	0.0190 (3)	
C2A	0.9351 (2)	0.41382 (10)	0.69739 (10)	0.0202 (3)	
H2AA	1.0832	0.4363	0.7185	0.024*	
C3A	0.7849 (2)	0.38121 (10)	0.74952 (9)	0.0194 (3)	
C4A	0.5674 (2)	0.34761 (10)	0.72071 (10)	0.0217 (3)	
H4AA	0.4688	0.3259	0.7579	0.026*	
C5A	0.4968 (2)	0.34643 (10)	0.63666 (10)	0.0201 (3)	
H5AA	0.3487	0.3232	0.6160	0.024*	
C6A	0.6411 (2)	0.37909 (9)	0.58198 (9)	0.0173 (3)	
C7A	0.4114 (2)	0.35695 (9)	0.45511 (9)	0.0179 (3)	
H7AA	0.2949	0.3322	0.4836	0.021*	
C8A	0.3686 (2)	0.36216 (9)	0.36702 (9)	0.0179 (3)	
C9A	0.1576 (2)	0.33220 (10)	0.32528 (10)	0.0189 (3)	
H9AA	0.0445	0.3081	0.3557	0.023*	
C10A	0.1101 (2)	0.33680 (10)	0.24159 (10)	0.0204 (3)	
H10A	−0.0344	0.3158	0.2153	0.024*	
C11A	0.2740 (2)	0.37246 (10)	0.19399 (9)	0.0174 (3)	
C12A	0.4886 (2)	0.40115 (10)	0.23598 (10)	0.0197 (3)	
H12A	0.6033	0.4239	0.2054	0.024*	
C13A	0.5328 (2)	0.39655 (10)	0.31967 (9)	0.0188 (3)	
H13A	0.6772	0.4170	0.3463	0.023*	
C14A	0.4024 (3)	0.40912 (13)	0.06081 (10)	0.0286 (3)	
H14A	0.4798	0.4733	0.0951	0.043*	
H14B	0.3383	0.4133	0.0051	0.043*	
H14C	0.5058	0.3597	0.0476	0.043*	
C15A	0.0107 (2)	0.34709 (12)	0.06894 (10)	0.0267 (3)	
H15A	−0.0982	0.3811	0.1074	0.040*	
H15B	−0.0213	0.2760	0.0576	0.040*	
H15C	0.0050	0.3615	0.0125	0.040*	
O1B	0.52861 (17)	0.06146 (8)	0.59158 (7)	0.0240 (2)	
H1B	0.449 (3)	0.0669 (15)	0.5502 (15)	0.038 (6)*	
O2B	0.62214 (19)	0.12261 (10)	0.91582 (8)	0.0358 (3)	
O3B	0.3155 (2)	0.17004 (9)	0.96784 (8)	0.0363 (3)	
N1B	0.4363 (2)	0.14478 (9)	0.90623 (9)	0.0259 (3)	
N2B	0.16151 (19)	0.12088 (8)	0.55922 (8)	0.0189 (2)	
N3B	−0.22861 (19)	0.09965 (9)	0.17216 (8)	0.0224 (3)	
C1B	0.4096 (2)	0.09763 (9)	0.66287 (9)	0.0182 (3)	
C2B	0.4863 (2)	0.10243 (10)	0.74690 (10)	0.0204 (3)	
H2BA	0.6231	0.0808	0.7562	0.024*	
C3B	0.3569 (2)	0.13995 (10)	0.81726 (9)	0.0208 (3)	
C4B	0.1556 (2)	0.17232 (10)	0.80674 (10)	0.0223 (3)	
H4BA	0.0703	0.1970	0.8563	0.027*	
C5B	0.0825 (2)	0.16755 (10)	0.72168 (10)	0.0199 (3)	
H5BA	−0.0540	0.1898	0.7131	0.024*	
C6B	0.2071 (2)	0.13050 (9)	0.64878 (9)	0.0175 (3)	
C7B	−0.0179 (2)	0.14324 (9)	0.53189 (9)	0.0183 (3)	
H7BA	−0.1218	0.1683	0.5741	0.022*	
C8B	−0.0682 (2)	0.13184 (9)	0.43956 (9)	0.0176 (3)	
C9B	−0.2682 (2)	0.15614 (9)	0.41343 (10)	0.0185 (3)	
H9BA	−0.3687	0.1806	0.4573	0.022*	
C10B	−0.3238 (2)	0.14565 (10)	0.32598 (9)	0.0189 (3)	
H10B	−0.4613	0.1626	0.3107	0.023*	
C11B	−0.1782 (2)	0.10990 (9)	0.25881 (9)	0.0180 (3)	
C12B	0.0245 (2)	0.08440 (10)	0.28521 (10)	0.0194 (3)	
H12B	0.1255	0.0597	0.2416	0.023*	
C13B	0.0761 (2)	0.09487 (10)	0.37254 (10)	0.0185 (3)	
H13B	0.2120	0.0768	0.3882	0.022*	
C14B	−0.0722 (3)	0.06855 (12)	0.10417 (10)	0.0280 (3)	
H14D	−0.0277	0.0051	0.1043	0.042*	
H14E	0.0570	0.1179	0.1167	0.042*	
H14F	−0.1406	0.0617	0.0458	0.042*	
C15B	−0.4374 (2)	0.12439 (12)	0.14568 (10)	0.0269 (3)	
H15D	−0.4564	0.1919	0.1810	0.040*	
H15E	−0.5567	0.0788	0.1556	0.040*	
H15F	−0.4398	0.1192	0.0825	0.040*	

### Atomic displacement parameters (Å^2^)

**Table:**

	*U*^11^	*U*^22^	*U*^33^	*U*^12^	*U*^13^	*U*^23^
O1A	0.0198 (5)	0.0421 (6)	0.0185 (5)	−0.0004 (4)	0.0012 (4)	0.0136 (4)
O2A	0.0304 (6)	0.0392 (6)	0.0236 (6)	0.0026 (5)	−0.0085 (5)	0.0109 (5)
O3A	0.0428 (7)	0.0556 (8)	0.0218 (6)	−0.0061 (6)	−0.0043 (5)	0.0239 (5)
N1A	0.0321 (7)	0.0237 (6)	0.0170 (6)	0.0046 (5)	−0.0035 (5)	0.0078 (5)
N2A	0.0214 (6)	0.0205 (5)	0.0140 (6)	0.0031 (4)	0.0001 (4)	0.0059 (4)
N3A	0.0194 (6)	0.0333 (6)	0.0164 (6)	0.0037 (5)	0.0002 (5)	0.0101 (5)
C1A	0.0206 (7)	0.0203 (6)	0.0166 (7)	0.0039 (5)	0.0027 (5)	0.0057 (5)
C2A	0.0195 (6)	0.0218 (6)	0.0189 (7)	0.0039 (5)	−0.0022 (5)	0.0057 (5)
C3A	0.0270 (7)	0.0183 (6)	0.0135 (7)	0.0045 (5)	−0.0016 (5)	0.0054 (5)
C4A	0.0259 (7)	0.0213 (6)	0.0192 (7)	−0.0005 (5)	0.0012 (6)	0.0093 (5)
C5A	0.0210 (6)	0.0212 (6)	0.0180 (7)	−0.0012 (5)	−0.0022 (5)	0.0075 (5)
C6A	0.0218 (6)	0.0146 (6)	0.0157 (7)	0.0033 (5)	−0.0010 (5)	0.0048 (5)
C7A	0.0205 (6)	0.0178 (6)	0.0164 (7)	0.0043 (5)	0.0024 (5)	0.0063 (5)
C8A	0.0209 (6)	0.0158 (6)	0.0168 (7)	0.0037 (5)	0.0003 (5)	0.0045 (5)
C9A	0.0205 (6)	0.0200 (6)	0.0170 (7)	0.0008 (5)	0.0014 (5)	0.0075 (5)
C10A	0.0168 (6)	0.0224 (6)	0.0212 (7)	0.0004 (5)	−0.0016 (5)	0.0066 (5)
C11A	0.0199 (6)	0.0194 (6)	0.0134 (7)	0.0052 (5)	−0.0004 (5)	0.0051 (5)
C12A	0.0184 (6)	0.0232 (6)	0.0180 (7)	0.0023 (5)	0.0020 (5)	0.0073 (5)
C13A	0.0175 (6)	0.0215 (6)	0.0171 (7)	0.0023 (5)	−0.0012 (5)	0.0055 (5)
C14A	0.0266 (8)	0.0452 (9)	0.0170 (7)	0.0020 (6)	0.0009 (6)	0.0150 (6)
C15A	0.0245 (7)	0.0421 (9)	0.0158 (7)	0.0054 (6)	−0.0025 (6)	0.0124 (6)
O1B	0.0217 (5)	0.0353 (6)	0.0183 (5)	0.0089 (4)	0.0041 (4)	0.0111 (4)
O2B	0.0342 (6)	0.0509 (7)	0.0255 (6)	0.0093 (5)	−0.0065 (5)	0.0158 (5)
O3B	0.0494 (7)	0.0472 (7)	0.0149 (6)	0.0183 (6)	0.0031 (5)	0.0094 (5)
N1B	0.0347 (7)	0.0252 (6)	0.0186 (7)	0.0039 (5)	−0.0035 (5)	0.0081 (5)
N2B	0.0219 (6)	0.0201 (5)	0.0150 (6)	0.0024 (4)	−0.0004 (4)	0.0062 (4)
N3B	0.0192 (6)	0.0340 (6)	0.0160 (6)	0.0058 (5)	0.0017 (5)	0.0099 (5)
C1B	0.0194 (6)	0.0189 (6)	0.0171 (7)	0.0007 (5)	0.0020 (5)	0.0073 (5)
C2B	0.0198 (6)	0.0213 (6)	0.0217 (7)	0.0026 (5)	−0.0014 (5)	0.0093 (5)
C3B	0.0284 (7)	0.0195 (6)	0.0141 (7)	−0.0006 (5)	−0.0029 (6)	0.0059 (5)
C4B	0.0284 (7)	0.0212 (6)	0.0172 (7)	0.0049 (5)	0.0034 (6)	0.0049 (5)
C5B	0.0215 (6)	0.0211 (6)	0.0179 (7)	0.0053 (5)	0.0009 (5)	0.0064 (5)
C6B	0.0206 (6)	0.0156 (6)	0.0169 (7)	0.0001 (5)	−0.0001 (5)	0.0066 (5)
C7B	0.0185 (6)	0.0174 (6)	0.0185 (7)	0.0008 (5)	0.0013 (5)	0.0054 (5)
C8B	0.0190 (6)	0.0161 (6)	0.0177 (7)	0.0000 (5)	−0.0006 (5)	0.0060 (5)
C9B	0.0199 (6)	0.0183 (6)	0.0174 (7)	0.0036 (5)	0.0028 (5)	0.0049 (5)
C10B	0.0179 (6)	0.0203 (6)	0.0195 (7)	0.0046 (5)	−0.0006 (5)	0.0069 (5)
C11B	0.0190 (6)	0.0183 (6)	0.0172 (7)	0.0005 (5)	−0.0012 (5)	0.0069 (5)
C12B	0.0174 (6)	0.0234 (6)	0.0186 (7)	0.0046 (5)	0.0033 (5)	0.0077 (5)
C13B	0.0162 (6)	0.0208 (6)	0.0203 (7)	0.0032 (5)	0.0005 (5)	0.0087 (5)
C14B	0.0273 (7)	0.0424 (9)	0.0168 (7)	0.0085 (6)	0.0036 (6)	0.0112 (6)
C15B	0.0269 (7)	0.0395 (8)	0.0171 (7)	0.0103 (6)	−0.0012 (6)	0.0109 (6)

### Geometric parameters (Å, º)

**Table:**

O1A—C1A	1.3532 (18)	O1B—C1B	1.3545 (17)
O1A—H1A	0.85 (2)	O1B—H1B	0.84 (2)
O2A—N1A	1.2339 (17)	O2B—N1B	1.2357 (18)
O3A—N1A	1.2318 (18)	O3B—N1B	1.2268 (18)
N1A—C3A	1.4566 (18)	N1B—C3B	1.4609 (18)
N2A—C7A	1.2853 (19)	N2B—C7B	1.2827 (18)
N2A—C6A	1.3966 (18)	N2B—C6B	1.4021 (18)
N3A—C11A	1.3596 (19)	N3B—C11B	1.3605 (19)
N3A—C15A	1.4542 (19)	N3B—C15B	1.4535 (18)
N3A—C14A	1.4611 (19)	N3B—C14B	1.4569 (18)
C1A—C2A	1.379 (2)	C1B—C2B	1.382 (2)
C1A—C6A	1.4184 (19)	C1B—C6B	1.4132 (19)
C2A—C3A	1.392 (2)	C2B—C3B	1.387 (2)
C2A—H2AA	0.9500	C2B—H2BA	0.9500
C3A—C4A	1.389 (2)	C3B—C4B	1.391 (2)
C4A—C5A	1.387 (2)	C4B—C5B	1.391 (2)
C4A—H4AA	0.9500	C4B—H4BA	0.9500
C5A—C6A	1.3985 (19)	C5B—C6B	1.3960 (19)
C5A—H5AA	0.9500	C5B—H5BA	0.9500
C7A—C8A	1.441 (2)	C7B—C8B	1.444 (2)
C7A—H7AA	0.9500	C7B—H7BA	0.9500
C8A—C9A	1.4047 (19)	C8B—C9B	1.4002 (19)
C8A—C13A	1.4083 (19)	C8B—C13B	1.4099 (19)
C9A—C10A	1.375 (2)	C9B—C10B	1.378 (2)
C9A—H9AA	0.9500	C9B—H9BA	0.9500
C10A—C11A	1.418 (2)	C10B—C11B	1.4135 (19)
C10A—H10A	0.9500	C10B—H10B	0.9500
C11A—C12A	1.4245 (19)	C11B—C12B	1.4239 (19)
C12A—C13A	1.371 (2)	C12B—C13B	1.372 (2)
C12A—H12A	0.9500	C12B—H12B	0.9500
C13A—H13A	0.9500	C13B—H13B	0.9500
C14A—H14A	0.9800	C14B—H14D	0.9800
C14A—H14B	0.9800	C14B—H14E	0.9800
C14A—H14C	0.9800	C14B—H14F	0.9800
C15A—H15A	0.9800	C15B—H15D	0.9800
C15A—H15B	0.9800	C15B—H15E	0.9800
C15A—H15C	0.9800	C15B—H15F	0.9800
			
C1A—O1A—H1A	102.7 (15)	C1B—O1B—H1B	101.7 (14)
O3A—N1A—O2A	122.88 (13)	O3B—N1B—O2B	123.00 (13)
O3A—N1A—C3A	118.47 (12)	O3B—N1B—C3B	118.68 (12)
O2A—N1A—C3A	118.63 (13)	O2B—N1B—C3B	118.32 (13)
C7A—N2A—C6A	122.31 (12)	C7B—N2B—C6B	122.21 (12)
C11A—N3A—C15A	120.42 (12)	C11B—N3B—C15B	120.50 (12)
C11A—N3A—C14A	121.04 (12)	C11B—N3B—C14B	121.35 (12)
C15A—N3A—C14A	117.87 (12)	C15B—N3B—C14B	118.08 (12)
O1A—C1A—C2A	120.56 (12)	O1B—C1B—C2B	120.39 (12)
O1A—C1A—C6A	117.96 (13)	O1B—C1B—C6B	118.22 (13)
C2A—C1A—C6A	121.48 (13)	C2B—C1B—C6B	121.39 (13)
C1A—C2A—C3A	117.87 (13)	C1B—C2B—C3B	117.73 (13)
C1A—C2A—H2AA	121.1	C1B—C2B—H2BA	121.1
C3A—C2A—H2AA	121.1	C3B—C2B—H2BA	121.1
C4A—C3A—C2A	122.61 (13)	C2B—C3B—C4B	123.07 (13)
C4A—C3A—N1A	119.16 (13)	C2B—C3B—N1B	118.07 (13)
C2A—C3A—N1A	118.22 (12)	C4B—C3B—N1B	118.87 (13)
C5A—C4A—C3A	118.76 (13)	C3B—C4B—C5B	118.20 (13)
C5A—C4A—H4AA	120.6	C3B—C4B—H4BA	120.9
C3A—C4A—H4AA	120.6	C5B—C4B—H4BA	120.9
C4A—C5A—C6A	120.78 (13)	C4B—C5B—C6B	120.85 (13)
C4A—C5A—H5AA	119.6	C4B—C5B—H5BA	119.6
C6A—C5A—H5AA	119.6	C6B—C5B—H5BA	119.6
N2A—C6A—C5A	129.15 (13)	C5B—C6B—N2B	128.86 (12)
N2A—C6A—C1A	112.36 (12)	C5B—C6B—C1B	118.77 (13)
C5A—C6A—C1A	118.49 (13)	N2B—C6B—C1B	112.37 (12)
N2A—C7A—C8A	122.61 (13)	N2B—C7B—C8B	122.64 (13)
N2A—C7A—H7AA	118.7	N2B—C7B—H7BA	118.7
C8A—C7A—H7AA	118.7	C8B—C7B—H7BA	118.7
C9A—C8A—C13A	117.62 (13)	C9B—C8B—C13B	117.45 (13)
C9A—C8A—C7A	120.11 (12)	C9B—C8B—C7B	120.10 (12)
C13A—C8A—C7A	122.28 (12)	C13B—C8B—C7B	122.44 (12)
C10A—C9A—C8A	121.78 (13)	C10B—C9B—C8B	122.06 (13)
C10A—C9A—H9AA	119.1	C10B—C9B—H9BA	119.0
C8A—C9A—H9AA	119.1	C8B—C9B—H9BA	119.0
C9A—C10A—C11A	120.89 (13)	C9B—C10B—C11B	120.62 (12)
C9A—C10A—H10A	119.6	C9B—C10B—H10B	119.7
C11A—C10A—H10A	119.6	C11B—C10B—H10B	119.7
N3A—C11A—C10A	121.71 (13)	N3B—C11B—C10B	121.70 (12)
N3A—C11A—C12A	121.19 (13)	N3B—C11B—C12B	120.93 (12)
C10A—C11A—C12A	117.09 (13)	C10B—C11B—C12B	117.37 (13)
C13A—C12A—C11A	121.23 (13)	C13B—C12B—C11B	121.09 (12)
C13A—C12A—H12A	119.4	C13B—C12B—H12B	119.5
C11A—C12A—H12A	119.4	C11B—C12B—H12B	119.5
C12A—C13A—C8A	121.36 (13)	C12B—C13B—C8B	121.41 (12)
C12A—C13A—H13A	119.3	C12B—C13B—H13B	119.3
C8A—C13A—H13A	119.3	C8B—C13B—H13B	119.3
N3A—C14A—H14A	109.5	N3B—C14B—H14D	109.5
N3A—C14A—H14B	109.5	N3B—C14B—H14E	109.5
H14A—C14A—H14B	109.5	H14D—C14B—H14E	109.5
N3A—C14A—H14C	109.5	N3B—C14B—H14F	109.5
H14A—C14A—H14C	109.5	H14D—C14B—H14F	109.5
H14B—C14A—H14C	109.5	H14E—C14B—H14F	109.5
N3A—C15A—H15A	109.5	N3B—C15B—H15D	109.5
N3A—C15A—H15B	109.5	N3B—C15B—H15E	109.5
H15A—C15A—H15B	109.5	H15D—C15B—H15E	109.5
N3A—C15A—H15C	109.5	N3B—C15B—H15F	109.5
H15A—C15A—H15C	109.5	H15D—C15B—H15F	109.5
H15B—C15A—H15C	109.5	H15E—C15B—H15F	109.5
			
O1A—C1A—C2A—C3A	179.99 (12)	O1B—C1B—C2B—C3B	−179.84 (12)
C6A—C1A—C2A—C3A	0.1 (2)	C6B—C1B—C2B—C3B	0.53 (19)
C1A—C2A—C3A—C4A	0.2 (2)	C1B—C2B—C3B—C4B	0.1 (2)
C1A—C2A—C3A—N1A	179.76 (11)	C1B—C2B—C3B—N1B	179.69 (12)
O3A—N1A—C3A—C4A	2.13 (19)	O3B—N1B—C3B—C2B	−174.00 (13)
O2A—N1A—C3A—C4A	−179.11 (13)	O2B—N1B—C3B—C2B	5.6 (2)
O3A—N1A—C3A—C2A	−177.50 (13)	O3B—N1B—C3B—C4B	5.6 (2)
O2A—N1A—C3A—C2A	1.26 (19)	O2B—N1B—C3B—C4B	−174.83 (13)
C2A—C3A—C4A—C5A	0.0 (2)	C2B—C3B—C4B—C5B	−0.6 (2)
N1A—C3A—C4A—C5A	−179.58 (12)	N1B—C3B—C4B—C5B	179.79 (12)
C3A—C4A—C5A—C6A	−0.5 (2)	C3B—C4B—C5B—C6B	0.5 (2)
C7A—N2A—C6A—C5A	−0.7 (2)	C4B—C5B—C6B—N2B	−179.40 (13)
C7A—N2A—C6A—C1A	179.33 (12)	C4B—C5B—C6B—C1B	0.1 (2)
C4A—C5A—C6A—N2A	−179.26 (13)	C7B—N2B—C6B—C5B	−3.0 (2)
C4A—C5A—C6A—C1A	0.7 (2)	C7B—N2B—C6B—C1B	177.45 (12)
O1A—C1A—C6A—N2A	−0.45 (17)	O1B—C1B—C6B—C5B	179.73 (11)
C2A—C1A—C6A—N2A	179.45 (12)	C2B—C1B—C6B—C5B	−0.6 (2)
O1A—C1A—C6A—C5A	179.58 (12)	O1B—C1B—C6B—N2B	−0.71 (17)
C2A—C1A—C6A—C5A	−0.5 (2)	C2B—C1B—C6B—N2B	178.93 (12)
C6A—N2A—C7A—C8A	−179.89 (11)	C6B—N2B—C7B—C8B	−178.80 (11)
N2A—C7A—C8A—C9A	−179.16 (12)	N2B—C7B—C8B—C9B	179.07 (13)
N2A—C7A—C8A—C13A	1.0 (2)	N2B—C7B—C8B—C13B	0.2 (2)
C13A—C8A—C9A—C10A	−0.6 (2)	C13B—C8B—C9B—C10B	−0.6 (2)
C7A—C8A—C9A—C10A	179.49 (12)	C7B—C8B—C9B—C10B	−179.44 (12)
C8A—C9A—C10A—C11A	−0.1 (2)	C8B—C9B—C10B—C11B	−0.3 (2)
C15A—N3A—C11A—C10A	−4.0 (2)	C15B—N3B—C11B—C10B	−0.7 (2)
C14A—N3A—C11A—C10A	−174.38 (13)	C14B—N3B—C11B—C10B	176.16 (13)
C15A—N3A—C11A—C12A	177.25 (13)	C15B—N3B—C11B—C12B	179.00 (13)
C14A—N3A—C11A—C12A	6.9 (2)	C14B—N3B—C11B—C12B	−4.1 (2)
C9A—C10A—C11A—N3A	−177.64 (13)	C9B—C10B—C11B—N3B	−179.46 (12)
C9A—C10A—C11A—C12A	1.2 (2)	C9B—C10B—C11B—C12B	0.80 (19)
N3A—C11A—C12A—C13A	177.30 (12)	N3B—C11B—C12B—C13B	179.83 (12)
C10A—C11A—C12A—C13A	−1.5 (2)	C10B—C11B—C12B—C13B	−0.43 (19)
C11A—C12A—C13A—C8A	0.8 (2)	C11B—C12B—C13B—C8B	−0.5 (2)
C9A—C8A—C13A—C12A	0.29 (19)	C9B—C8B—C13B—C12B	0.94 (19)
C7A—C8A—C13A—C12A	−179.84 (12)	C7B—C8B—C13B—C12B	179.79 (12)

### Hydrogen-bond geometry (Å, º)

**Table:**

*D*—H···*A*	*D*—H	H···*A*	*D*···*A*	*D*—H···*A*
O1*A*—H1*A*···N2*A*	0.85 (2)	1.99 (2)	2.5860 (16)	126 (2)
O1*A*—H1*A*···O1*A*^i^	0.85 (2)	2.41 (2)	2.8738 (16)	114.7 (19)
O1*B*—H1*B*···N2*B*	0.84 (2)	1.993 (19)	2.5896 (16)	127.5 (19)
O1*B*—H1*B*···O1*B*^ii^	0.84 (2)	2.45 (2)	2.8853 (15)	113.7 (17)
C7*B*—H7*BA*···O1*B*^iii^	0.95	2.58	3.1870 (17)	122
C15*A*—H15*C*···O3*A*^iv^	0.98	2.59	3.3989 (19)	141

Symmetry codes: (i) −*x*+2, −*y*+1, −*z*+1; (ii) −*x*+1, −*y*, −*z*+1; (iii) *x*−1, *y*, *z*; (iv) *x*−1, *y*, *z*−1.
